# Antibiotics’ resistance profile of pathogens isolated from fish products sold in the city of Bangangté, Cameroon: Aqueous extracts from spices’ formulations used as accompanying soup of braised fish as antimicrobial alternative

**DOI:** 10.1016/j.heliyon.2024.e40716

**Published:** 2024-11-28

**Authors:** Hippolyte Tene Mouafo, Majeste Mbiada Pahane, Paul Alain Nana, Hermes Tsabet, Alphonse Tegang Sokamte, Thierry Ngangmou Noumo, Ingrid Cecile Djuikoue, Agbor Michael Ashu, François Tchoumbougnang

**Affiliations:** a Centre for Food, Food Security and Nutrition Research, Institute of Medical Research and Medicinal Plant Studies, PoBox 13033, Yaoundé, Cameroon; b Department of Processing and Quality Control of Aquatic Products, Institute of Fisheries and Aquatic Sciences at Yabassi, University of Douala, PoBox 7236, Douala, Cameroon; c Agriculture and Food Safety Association (AFSA), Cameroon; d Department of Oceanography, Institute of Fisheries and Aquatic Sciences at Yabassi, University of Douala, PoBox 7236, Douala, Cameroon; e Higher Institute of Health Science, Université des Montagnes, PoBox, 208 Bangangté, Cameroon; f Department of Food Engineering and Quality Control, University Institute of Technology, University of Ngaoundéré, PoBox 454, Ngaoundéré, Cameroon; g Department of Food Science and Technology, College of Technology, University of Bamenda, PoBox 39, Bambili, Cameroon

**Keywords:** Raw fish, Braised fish, Pathogens, MAR index, Spice formulation, Antimicrobial activity

## Abstract

The present study aimed to evaluate the antimicrobial activity of aqueous extracts of the mixture of spices used as accompanying soup of braised fish against multidrug resistant (MDR) bacteria isolated from raw and braised fish collected in the city of Bangangté, Cameroon. A survey was conducted in the city of Bangangté to diagnose the braising fish processes. Pathogens were isolated from raw and braised fish samples collected in fish farms and selling points, and their susceptibility to 16 commonly used antibiotics was tested using the Kirby-Bauer disk diffusion method. The aqueous extracts of spices’ formulations used as accompanying soup of braised fish were tested against MDR isolates using the well diffusion method. The results revealed that 13 spices are used in the preparation of accompanying soups of braised fish. Three formulations were identified with mbongo, clove and prekeses as the main differential spices. These formulations were either heated at 100 °C/30 min (process 1) or not (process 2) before being served to consumers’. Fifty-five strains belonging to six species (*Salmonella* Typhi, *Salmonella* Paratyphi, *Escherichia coli*, *Escherichia vulneralis*, *Staphylococcus aureus* and *Staphylococcus* spp.) were isolated from the different samples. Forty-four isolates were resistant to several antibiotics with Multiple Antibiotic Resistance indexes ranging from 0.18 to 0.81. The aqueous extracts of all the formulations were active against the MDR isolates independent of the formulation and preparation process, with inhibition diameters varying significantly (p < 0.05) from one isolate to another. Heated extracts were less active than the unheated ones and the most active extract was from the formulation including clove. The strains *E. coli* and *E. vulneralis* were most resistant to the different extracts whereas *S.* Typhi, *S. aureus*, *Staphylococcus* spp. and *S.* Paratyphi A, were more sensitive. This study evidenced that fish products from Bangangté city contain MDR pathogens and demonstrated that the formulations of spices used as accompanying soup for braised fish are active against these MDR pathogens. It suggests their potential use as strategy to combat the antimicrobial resistance phenomena associated to foodborne gastroenteritis pathogens. It also suggests that more attention should be paid to the use of antibiotics in fish farming activities.

## 1 Introduction

Aquaculture was introduced in Cameroon since 1940 due to a deficiency of animal protein sources to satisfy the consumers’ demand. It has made significant progress over the last ten years and is practiced in fresh water and ponds [1]. Nowadays, the fish production estimated at 400,000 tons in 2015 is still insufficient to meet the market demand due to the growing population (2.8 per year) and the rapid urbanization which has consequently led to a significant increase in fish prices in Cameroon [1]. In order to meet increasing demand of consumers, antibiotics are being used abusively in fish farming, which could lead to the presence of antibiotic residues in fish [[2], [3], [4]]. The exposition of fish microflora to these antibiotic residues might result in the development of resistances [5]. Multidrug resistant (MDR) pathogens were isolated from raw fish farmed in the city of Yaoundé, Cameroon [6] as well as in braised fish sold the city of Bangangté, Cameroon [7]. Thus, the consumption of these fish and derived products could lead to foodborne diseases that could not be treated with conventional antibiotics. Among the most incriminated MDR pathogens in foodborne diseases, strains of *S. aureus*, *E. coli*, and *Salmonella* spp. figure out. Indeed, *S. aureus* is a major human and animal pathogens [8]. The infection mechanism of *S. aureus* is due to the ability of the pathogens to produce virulence factors such as enterotoxins, exotoxins, surface proteins, biofilms, exfoliative toxins, hemolysins, surface proteins, pathogenicity islands, adhesins and integrative conjugative elements [9,10]. The expression of genes encoding for these factors allows *S. aureus* to attach to tissue, penetrate the immune system, and cause toxicity [10,11]. In addition, *S. aureus* harbors genes responsible for resistance against several antibiotics including methylicin, chloramphenicol, penicillin, piperacillin, gentamicin, levofloxacin and others [8,12]. The pathogenic potential of *E. coli* is related to their virulence genes [13]. Based on these virulence factors, they are Enterohaemorrhagic *Escherichia coli* (EHEC) harboring genes *eae, Stx1* and *Stx2*, Enteropathogenic *Escherichia coli* (EPEC) harboring genes *f17A, cnf, papEF, Afa/draBC, fyua, clbN, hlyf, kpsMT*(K1), *hlyA*, and *Sfa/focDE*, Enteroinvasive *Escherichia coli* (EIEC) harboring gene *Ipah*, Enteroaggregative *Escherichia coli* (EAEC) harboring genes *AAprobe, aap* and *aggr* [14,15]. According to D’Onofrio et al. [16], some strains of *E. coli* harbor virulence genes (kpsMII_K23 and K1 capsule group 2) that are associated with human dead while factors such as microcins, toxins, and fimbriae are responsible of the severity of diseases. Albeit being highly pathogens, strains of *E. coli* contain genes encoding for antibiotic efflux (*TolC*, *emrR*, *evgA*, *qacEdelta1*, *H-NS*, *cpxA*, and *mdtM*), antibiotic inactivation (*aadA5*, *mphA*, and *CTX-M-15*), and antibiotic drug replacement (*sul1* and *dfrA14*) [15]. *Salmonella* is among the main bacteria incriminated worldwide in foodborne diseases. It harbors antibiotic resistances genes including *bla*_*TEM-1*_ and *bla*_*SHV*_ for β-lactams, *aadA1* and *Aac(3)-Ia* for aminoglycosides, *sul1* for sulfonamides and *parC* for quinolones [17]. Its high pathogenicity is related to the interaction between these antibiotic resistance genes and virulence factors like fimbriae, capsule, adhesins, plasmids, enterotoxins and virulence islands [18,19]. Through that interaction, the adhesion and invasion mechanism are activated leading to infection [20].

Hence, multidrug resistance has been increased all over the world that is considered a public health threat [[21], [22], [23], [24]]. Several recent investigations reported the emergence of multidrug-resistant bacterial pathogens from different origins that increase the necessity of the proper use of antibiotics [7,12,24,25]. Besides, the routine application of the antimicrobial susceptibility testing to detect the antibiotic of choice as well as the screening of the emerging MDR strains [26,27]. It appears therefore interesting to find natural compounds endowed with antimicrobial activities able to overcome the multidrug resistance microorganisms. In Cameroon, fish is highly consumed in its braised form accompanied with a spicy soup prepared from a mixture of several spices [28,29]. Several researches have highlighted the antimicrobial activities of these spices taken individually against Gram positive and negative bacteria [30,31] as well as microscopic fungi [32,33]. However, there is a gap in the literature regarding the antimicrobial activity of the combination of these different spices. We therefore hypothesize that the accompanying spicy soup of braised fish might deserve high antimicrobial activity. In order to contribute to the reduction of the phenomena of multidrug resistance of microorganisms involved in foodborne diseases, the present study was designed. The objective of this study was to: i) assess the antibiotic resistance profile of some pathogens isolated from raw fish (flesh and intestine) and its derived products (braised fish) sold in the city of Bangangté, and ii) evaluate the antimicrobial activity of mixtures of selected Cameroonian spices used as accompanied soup of braised fish against the MDR strains of pathogens.

## 2 Materials and methods

### 2.1 Study area and period

The study was carried out from September to November 2020 in the City of Bangangté (5° 15’N and 10° 50’ E), Department of Nde, Western Region of Cameroon. The city of Bangangté was chosen because braised fishes are widely sold and consumed by the local inhabitants [7].

### 2.2 Study design and population

A descriptive cross-sectional study was conducted in the city of Bangangté. Braising fish vendors were randomly selected in the city of Bangangté. This study was approved by the National Ethics Committee of Cameroon under No. 2020/164/AED/UDM/CUM. Besides, approval of the administrative authorities in charge of the city was also obtained. The participants involved in the study were volunteers and they signed a free and informed consent form.

### 2.3 Questionnaire administration and data collection

A survey on the braising fish practice was carried out with the help of a structured questionnaire and an observation check-list. Using the convenience sampling method, braising fish vendors was selected and interviewed in their different working places. The questionnaire administrated to these vendors seek information concerning: the socio demographic parameters, the choice of fish species and the braising process, the type of spices used and their composition, the treatment of fish after embering, the fate of braised fish which has not been sold and finally the selling conditions. The observation check-list established following the guide of Good Hygiene and Manufacturing Practices [34] was used to assess the level of hygiene of the braising fish vendors.

### 2.4 Samples collection

For raw fish samples collection, the two major fish farms present in the city of Bangangté were selected. Associated to these two fish farms, the largest fish stores where braising fish vendors always collected raw fish when the ones from farms are not available was also selected as selected as sampling site [7]. In the three sampling sites, ten fishes of approximately 500 g each were randomly collected, packaged in sterile bags, labelled, introduced into an ice box containing cold accumulators and transported to the laboratory.

### 2.5 Samples processing

Upon arrival at the laboratory, under aseptic conditions, the fish samples were descaled, degutted, rinsed with sterile distilled water and fleshes were divided in two groups. The first group made of 5 entire raw fish per sample was braised at the laboratory following the process described by the braising fish vendors while the second group was constituted of the 5 raw fish fleshes per sample. Besides, the guts from each sample were also collected. The three types of samples (raw fish fleshes, braised fishes and guts) were submitted to microbiological analysis.

### 2.6 Microbiological analyses

#### 2.6.1 Samples preparation

The different samples were prepared following the method ISO 6887-2 [35]. Under aseptic conditions, the five repetitions from each sample were mixed, crushed and homogenized. From the mixture, 25 g were taken and transferred into a sterile Erlenmeyer. 225 mL of sterile peptone water (LiofilChem, Roseto degli Abruzzi, Italy) were added and the mixture was homogenized, left at room temperature for 30 min and serially diluted (10^−1^ to 10^−6^).

#### 2.6.2 Isolation of *Escherichia coli*

*E. coli* strains were isolated from the samples using the dilution and surface inoculation method [36]. Briefly, 0.1 mL of the different dilutions was introduced into a Petri dish containing 20 mL of sterile Eosin Methylene Blue agar (EMB, LiofilChem, Roseto degli Abruzzi, Italy) and spread. The Petri dishes were incubated under aerobic conditions at 44 °C for 48 h. Metal green colonies appearing on EMB agar were considered as *E. coli*. Individual metal green colonies was picked up with an inoculation loop and transferred into a newly prepared EMB by striking and the plate was incubated (44 °C/24 h). This operation was repeated two times to insure the purification of isolates. Then these isolates were kept for identification.

#### *2.6.3* *Isolation of Staphylococcus* spp.

Strains belonging to the genus *Staphylococcus* were isolated from the different samples following the normalized method ISO 6888-2 [37]. In the protocol, 0.1 mL of the different dilutions was surface inoculated into sterile Petri dish containing 20 mL of sterile Mannitol Salt Agar (MSA, LiofilChem, Roseto degli Abruzzi, Italy). The plates were incubated at 37 °C for 48 h under aerobic conditions. Presumptive small, round, pigmented in golden yellow colonies and surrounded by a yellow halo were transferred twice into sterile MSA agar plates. The isolates were stored for identification.

#### *2.6.4* *Isolation of Salmonella* spp.

The method ISO 6579-1 [38] was used for the isolation of *Salmonella* spp. from the different samples. The homogenized mixture made of 25 g of sample and 225 mL of peptone water was incubated for 16 h at 37 °C under aerobic conditions. Then, 1 mL of the suspension was transferred into a tube containing 10 mL of sterile Selenite Cysteine broth (LiofilChem, Roseto degli Abruzzi, Italy) and incubated for 24 h at 37 °C. Thereafter, one loopful of each enrichment broth was streaked onto sterile Petri dish containing 20 mL of *Salmonella* and *Shigella* agar (SS, LiofilChem, Roseto degli Abruzzi, Italy). The Petri dishes were incubated under aerobic conditions at 37 °C for 24 h. Uncolored colonies with black centers appearing on the Petri dish after incubation were purified through subculture twice on sterile SS agar. The purified isolates were kept for characterization.

#### 2.6.5 Identification of the isolates

The pure colonies of the different isolates were characterized using microscopic (colony shape and color), microscopic (Gram staining), cultural (growth at different temperatures, pH and salinity) and biochemical (catalase, coagulase, urease, methyl red, indole, oxidase) tests. The different tests were performed according to Bergey’s manual of systematics of archaea and bacteria [39]. Further characterization of the isolates was performed through their Analytical Profile Index (API). Experiments were performed according to the manufacturers’ instructions. The results gathered from API galleries were computerized using the software Apident 2.0 (BioMérieux, Craponne, France). The identities obtained from the Apident software were confirmed online (https://apiweb.biomerieux.com).

### 2.7 Antibiotics resistance profile of the different isolates

The antibiotics resistance profile of the different isolates obtained in this study was assessed following the method described by the Clinical and Laboratory Standards Institute [40]. A total of 15 commonly used antibiotics belonging to 10 classes were tested against the different isolates. They were: Amoxicillin (AMX, 30 μg), Oxacillin (OX, 5 μg) and Penicillin (PEN, 6 μg) of penicillins class, Amoxicillin-clavulanic acid (AMC, 30 μg) of Beta-lactams class, Ciprofloxacin (CIP, 5 μg) of fluoroquinolones class, Cotrimoxazole (SXT, 25 μg) of sulfonamides class, Cefotaxime (CTX, 10 μg), Ceftazidime (CAZ, 10 μg) and Cefuroxime (CXM, 30 μg) of Cephalosporins class, Cefoperazone (CFP, 30 μg), Erythromycin (ERY, 10 μg) of macrolides class, Gentamycin (GEN, 10 μg) and Kanamycin (KAN, 30 μg) of aminoglycosides class, Imipenem (IMP, 10 μg) of carbapenems class, Nitrofurantoin (NIF, 300 μg) of Nitrofurans class, and Tetracycline (TE, 30 μg) of tetracyclines class. The different isolates were cultured in nutrient broth (LiofilChem, Roseto degli Abruzzi, Italy) at 37 °C for 16 h. Then the cultures were centrifuged at 6000 g, 20 min, 4 °C (Rotofix 32 A, Hettich Zentrifugen, Tuttlingen, Germany) and cells were collected, washed with sterile phosphate saline buffer (PBS, pH 7.2), resuspended in PBS and their loads adjusted to 5 × 10^5^ CFU/mL [41]. The tests were performed in Muller Hinton agar (MH, LiofilChem, Roseto degli Abruzzi, Italy) following the method described by Pahane et al. [7]. Briefly, 0.1 mL of inoculum was spread at the surface of sterile MH agar and the Petri dishes were left on the bench (25 ± 1 °C) for 30 min. Then, six disks of the antibiotics were carefully placed in the Petri dishes followed with incubation at 37 °C for 24 h. After incubation, the diameters of the clearing zone surrounding each antibiotic disk were measured. Using the manual of the Clinical and Laboratory Standards, the isolates were classified based on their inhibition diameters as susceptible, intermediate or resistant [40]. The Multiple Antibiotic Resistance (MAR) index of the different isolates were determined [7]. The risk associated with the source from which the antibiotic resistance strain was isolated, was characterized following the method of Krumperman [42].

### 2.8 Screening of antimicrobial activity of the aqueous extracts from the spices’ formulations

#### 2.8.1 Preparation of extracts

Three different formulations of spices and two cooking processes were identified from the survey. The six samples of formulations were extracted using the method of Mouafo et al. [41] with some modifications. In the protocol, the formulation (mixture of several spices) was diluted with water at a ratio 1:1 (w/v) and the extraction process (maceration) was carried out at room temperature for 12 h under agitation of 800 rpm (Lab-line Pyro-multi Magnestir 1263-1, San Francisco, USA). The mixture was centrifuged (4000 g, 20 min) and the supernatant was collected. The supernatant was then filtered (Whatman N°4) and the filtrate was oven-dried at a temperature of 60 °C for 3 days. The dried extract was used to prepare a solution at 1 mg/mL.

#### 2.8.2 Inoculum preparation

Inoculums of young cells (16 h) from the different isolates were prepared following the method of Mouafo et al. [41] as previously described and their loads were adjusted to 5 × 10^5^ CFU/mL.

#### 2.8.3 Screening of antimicrobial activity of the extracts

The protocol of the well diffusion method of National Committee for Clinical Laboratory Standards described by Mouafo et al. [43] was used to screen the antimicrobial activity of the different extracts. Briefly, 0.1 mL of the different inoculums (0.5 McF) was spread at the surface of sterile MH agar poured into Petri dish. To allow the suspension to dry, the Petri dishes were left for 1 h on the bench at room temperature with lid closed. Then, six wells (diameter of 6 mm) were digged and 25 μL of the extracts were added in each well. The Petri dishes were left at 4 °C for 4 h and incubated at 37 °C for 24 h. The Petri dishes were observed for the zone of inhibition which is suggested by the clear area around the well. The diameters of inhibition zones were measured and recorded.

### 2.9 Statistical analysis

The data collected from the investigation survey were submitted to descriptive statistics and they were summarized as frequencies and percentages. For experiments carried out in the laboratory, all tests were performed in at least three repetitions. The results were expressed as means ± standard deviation. The means were compared using the Duncan Multiple range test. Significant difference following one-way analysis of variance (ANOVA) was set at p < 0.05 with Statgraphic Centurion XVI version 16.1.18 (StatPoint Technologies, Inc., Virginia, USA).

## 3 Results

The phenomena of multidrug resistance is in rise worldwide. In view of bringing solutions to face that issue, research are directed towards natural products endowed of antimicrobial activity. In this study we hypothesized that the accompanying spicy soup of braised fish made of a mixture of several spices might deserve high antimicrobial activity against MDR pathogens responsible of foodborne diseases. Our goal was to develop an eco-friendly and sustainable antimicrobial alternative against MDR pathogens using commonly consumed spices. For that, a survey was conducted to identify the formulations of accompanying spicy soup of braised fish; isolate and identify from raw and braised fish the main pathogens associated with foodborne diseases; determine the antibiotic resistance profile of these pathogens; and assess the antimicrobial activity of the identified spicy soup formulations on the MDR pathogens.

### 3.1 Socio-demographic characteristics of the study population

The braising fish activity was exclusively carried out by women as presented in Table 1. These vendors at 65 % were aged around 30 years old and above. Their level of education varied between the primary (40 %) and the secondary (60 %).

**Table 1:** Socio-demographic parameters of the population.

Parameters		Frequency	Percentages
Gender	Male	0	0
	Female	25	100
Age	15–20 years	3	12
	21–30 years	6	24
	31–50 years	15	60
	50 and more	1	4
Education level	No formal education	0	0
	Primary	10	40
	Secondary	15	60
	University	0	0

N = 25 participants.

### 3.2 Braising fish activity

The braising fish activity was practiced at full time by 85 % of participants for which it represents the main financial income. The raw fish used by vendors were originated from fish farms (60 %) and fish shops (40 %). Bar (90 %), mackerel (95 %), carp (86 %) and thon (15 %) were the species of fish braised and sold by the participants. The highest score recorded with mackerel could arise from its cheap price and thus its high demand.

### 3.3 Spicy soup preparation process

It emerges from this results that, a total of 13 different spices were used by fish vendors as the constituents for the formulation of the spicy soup. They were: mbongo (bark of garlic tree), alligator pepper (*Xylopia aethiopica*), cloves, prekeses, country onions, onion, African nutmeg, white and black pepper, garlic, ginger, njansang (*Ricinodendron heudelotii* seeds) and cumin seeds. The number of spices used in the formulation of the spicy soups varies from one vendor to another. Amongst the different spices, the most used by all fish vendors were onions (100 %) followed by white pepper (96 %). Njansang, garlic and country onions with a score of 90 % each, as well as African nutmeg and prekeses with the score of 80 % each, were also used in huge amount. However, they were not included in the formulation of the spicy soup by all vendors. 78 % of participants declared using clove, black pepper and cumin seeds in their formulation while ginger, mbongo and alligator pepper were used at 68 % each.

**Fig. 1:**
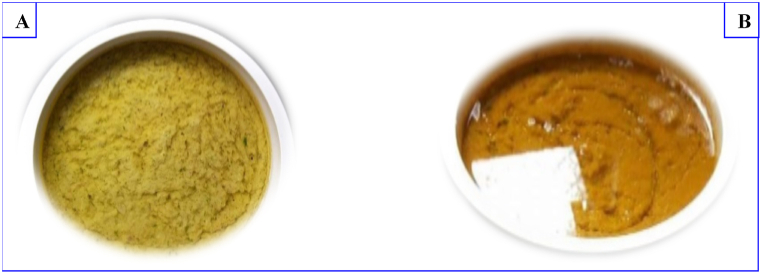
Uncooked (A) and cooked (B) formulation of spices used as accompanying soup for braised fish.

Fresh spices are cleaned with water, cut and introduced into a box. Garlic and onions are cleaned and introduced in the same box. Dry spices are directly added in the box. The mixture is ground and seasoned with salt and taste enhancing agent such as sodium glutamate. Then, oil is added and the mixture is homogenized. The spicy soup obtained is served to customers in raw form or after cooking for 30 min at 100 °C. Fig. 1 (A and B) show the uncooked (raw) and cooked spices’ formulation used as accompanying soup for braised fish. The main difference was the color that is yellow green for the uncooked formulation and brown for the cooked formulation.

With regards to the composition of the spices’ formulations, three different formulas were observed. These three formulations had as distinctive spices: clove, mbongo and prekeses. Taking into consideration the fact that these formulations were consumed raw (uncooked) and cooked, a total of six different formulations of spices were recorded. According to the participants, the prepared spicy soups can be used during 7 days (19 %), 3 days (43 %), 2 days (29 %) or 1 day (9 %) depending on the quantity prepared and the preparation process.

### 3.4 Microbiological profile of the different fish samples

As observed in Table 2, the 55 strains isolated from the different fish samples belonged to three genera: *Escherichia, Salmonella*, and *Staphylococcus.* From these three genera, five species of microorganisms were identified. They were *Escherichia coli, Escherichia vulneralis, Salmonella* Typhi, *Salmonella* Paratyphi A*, Staphylococcus aureus* and *Staphylococcus* spp.

**Table 2:** Prevalence and profile of microorganisms isolated from the different fish samples collected in three sites.

Sampling sites	Genus	Species	Number of isolates	Numbers and proportions of isolates per type of products		
				Raw flesh	Braised flesh	Gills
Fish farm A						
	*Escherichia*	*E. coli*	10	2 (20 %)	3 (30 %)	5 (50 %)
		*E. vulneralis*	0	0 (0 %)	0 (0 %)	0 (0 %)
	*Salmonella*	*S.* Typhi	4	1 (25 %)	2 (50 %)	1 (25 %)
		*S.* Paratyphi	4	4 (100 %)	0 (0 %)	0 (0 %)
	*Staphylococcus*	*S. aureus*	0	0 (0 %)	0 (0 %)	0 (0 %)
		*Staphylococcus* spp.	5	3 (60 %)	0 (0 %)	2 (40 %)
Fish farm B						
	*Escherichia*	*E. coli*	8	2 (25 %)	3 (37.50 %)	3 (37.50 %)
		*E. vulneralis*	0	0 (0 %)	0 (0 %)	0 (0 %)
	*Salmonella*	*S.* Typhi	5	1 (20 %)	2 (40 %)	2 (40 %)
		*S.* Paratyphi	0	0 (0 %)	0 (0 %)	0 (0 %)
	*Staphylococcus*	*S. aureus*	6	1 (16.67 %)	2 (33.33 %)	3 (60 %)
		*Staphylococcus* spp.	4	3 (75 %)	1 (25 %)	0 (0 %)
Fish shops						
	*Escherichia*	*E. coli*	0	0 (0 %)	0 (0 %)	0 (0 %)
		*E. vulneralis*	3	0 (0 %)	0 (0 %)	3 (100 %)
	*Salmonella*	*S.* Typhi	6	2 (33.33 %)	2 (33.33 %)	2 (33.33 %)
		*S.* Paratyphi	0	0 (0 %)	0 (0 %)	0 (0 %)
	*Staphylococcus*	*S. aureus*	0	0 (0 %)	0 (0 %)	0 (0 %)
		*Staphylococcus* spp.	0	0 (0 %)	0 (0 %)	0 (0 %)

Table 2 presents the frequency of occurrence of microorganisms isolated from the different fish samples collected in the three sites. In fish farm A, four species were identified. In raw flesh samples, the most isolated species were *S.* Paratyphi (04 isolates), *Staphylococcu*s spp. (03 isolates), *E. coli* (02 isolates) and the least isolated one was *S.* Typhi (01 isolate). In braised flesh samples, only two species were found. They were *E. coli* (03 isolates) and *S.* Typhi (02 isolates). The species *E. vulneralis*, *S.* Paratyphi, *S. aureus* and *Staphylococcus* spp. were not found. Isolates belonging only to the species *E. coli* (05 isolates), *Staphylococcus* spp. (02 isolates) and *S.* Typhi (01 isolate) were obtained from gill samples.

The samples collected from the fish shop showed the lowest microbial diversity (Table 2). The 09 strains isolated from the different samples were belonged to only two species (*S.* Typhi and *E. vulneralis*) independently of the types of products analyzed. Amongst these strains, 03 isolates belonging to *E. vulneralis* species were from gill samples and the rest of 06 isolates belonging to *S.* Typhi species were from raw flesh (02 isolates), braised flesh (02 isolates) and gill (02 isolates) samples.

*E. vulneralis* and *S.* Paratyphi were not detected in the samples from the fish farm B (Table 2). 3 isolates belonging to *E. coli* species were found in both braise flesh and gill samples while only 2 isolates were found in raw flesh samples. The same phenomenon was observed with the species *S.* Typhi as equal isolates (02) were present in braised flesh and gill samples. Only one isolate of *S.* Typhi was found in the raw flesh samples. With regards to the species *S. aureus*, 3 isolates were found in gill samples, 2 isolates in braised flesh and 1 isolate in raw flesh samples. Amongst the 04 isolates belonging to *Staphylococcus* spp., 3 were isolated from raw flesh samples and 01 form braised flesh samples.

### 3.5 Antibiotics susceptibility profile of the different isolates

The antibiotics which retained their antibacterial activity against the tested pathogens were gentamycin, imipenem, ciprofloxacin and amoxiclave with respective percentages of sensitivity of 81.81, 79.54, 68.19 and 61.37 %. The most active antibiotic against all the tested pathogens was gentamycin with 81.81 % of sensitivity (Table 3).

**Table 3:** Antibiotics resistance profile of the pathogens isolated from different fish samples sold in the city of Bangangté.

Test strains	Antibiotic susceptibility																MAR index
	AMC	SXT	GEN	AMX	CIP	KAN	CFP	NIF	CXM	TE	IMP	CTX	CAZ	PEN	ERY	OX	
*E. coli* A1a	R	R	S	R	R	R	R	S	R	R	S	R	R	I	I	I	0.62
*E. coli* A1c	S	S	S	S	S	S	R	S	R	S	S	R	I	S	S	S	0.18
*E. coli* A3a	R	S	S	S	S	S	R	S	R	S	S	S	R	I	I	I	0.25
*E. coli* A3b	R	S	S	S	S	S	R	S	R	S	S	S	R	I	I	I	0.25
*E. coli* A3c	R	S	S	R	S	R	R	R	R	R	S	S	R	I	I	I	0.50
*E. coli* A2c	R	S	S	S	S	R	R	S	R	S	S	S	R	I	I	I	0.31
*E. coli* A2d	R	S	S	S	S	R	R	S	R	S	S	S	R	I	I	I	0.31
*E. coli* B1a	R	R	S	R	S	R	S	S	R	R	S	R	R	I	I	I	0.50
*E. coli* B1b	R	R	R	R	S	R	R	S	R	R	S	R	R	I	I	I	0.56
*E. coli* B2c	R	R	S	S	R	R	R	R	R	R	S	R	R	I	I	I	0.62
*E. coli* B2d	R	S	S	S	S	S	S	S	S	R	S	S	R	I	I	I	0.18
*S.* Typhi A1c	R	S	S	S	R	R	S	S	R	S	S	R	R	I	I	I	0.37
*S.* Typhi A1d	R	S	S	S	R	R	S	S	R	S	S	R	R	I	I	I	0.37
*S.* Typhi A3b	R	R	R	R	R	R	R	R	R	R	R	S	R	I	I	I	0.68
*S.* Typhi A3a	R	R	S	R	S	R	S	R	R	S	S	R	R	I	I	I	0.50
*S.* Typhi A3c	R	S	S	R	R	R	R	S	R	R	S	R	R	I	I	I	0.56
*S.* Typhi A2a	R	R	S	S	S	R	R	R	R	R	S	R	R	I	I	I	0.56
*S.* Typhi A2b	R	R	R	S	S	R	R	R	R	R	S	R	R	I	I	I	0.56
*S.* Typhi A2c	R	R	S	S	S	R	R	S	R	R	R	R	R	I	I	I	0.56
*S.* Typhi A2d	R	R	S	R	R	R	S	R	R	R	R	R	R	I	I	I	0.68
*S.* Typhi B1c	R	R	S	R	S	R	R	R	R	R	S	R	R	I	I	I	0.62
*S.* Typhi B1d	R	R	S	R	S	R	R	R	R	R	S	R	R	I	I	I	0.62

Test strains	Antibiotic susceptibility																MAR index
	AMC	SXT	GEN	AMX	CIP	KAN	CFP	NIF	CXM	TE	IMP	CTX	CAZ	PEN	ERY	OX	
*S.* Typhi B2c	R	R	R	S	S	R	R	R	R	S	S	R	R	I	I	I	0.50
*S.* Typhi B2d	R	R	S	S	S	R	R	R	R	S	S	R	R	I	I	I	0.50
*S.* Typhi C1a	R	R	S	S	S	R	R	R	R	R	R	R	R	I	I	I	0.62
*S.* Typhi C1c	R	R	S	S	S	S	S	S	R	R	S	S	R	I	I	I	0.31
*S.* Typhi C1b	R	R	S	S	S	R	R	R	R	R	R	R	R	I	I	I	0.62
*Staphylococcus* spp. B1c	R	R	S	S	S	S	R	R	R	R	S	R	R	S	R	S	0.56
*Staphylococcus* spp. B1d	R	R	S	S	S	S	R	R	R	R	S	R	R	S	R	S	0.56
*Staphylococcus* spp. B2b	S	R	S	S	R	S	R	R	R	S	S	R	R	S	R	S	0.56
*Staphylococcus* spp. B2d	R	S	S	S	S	R	R	S	R	R	S	R	R	I	I	I	0.43
*Staphylococcus* spp. A1a	S	S	S	S	S	S	R	S	R	S	S	R	I	S	S	S	0.18
*Staphylococcus* spp. A1b	R	R	S	R	R	R	R	R	R	R	R	R	R	I	I	I	0.75
*Staphylococcus* spp. A1d	R	R	S	S	S	R	S	R	R	R	S	S	I	R	R	R	0.56
*Staphylococcus* spp. A3a	R	R	S	S	S	S	R	R	R	R	S	R	R	R	S	R	0.62
*Staphylococcus* spp. A3b	R	S	S	S	S	R	S	S	R	S	S	R	R	S	S	R	0.37
*Staphylococcus* spp. A3c	R	S	S	R	S	R	R	R	R	R	S	S	R	I	I	I	0.50
*S. aureus* B1a	R	S	S	S	R	S	R	R	R	R	S	R	R	I	I	I	0.50
*S. aureus* C1a	R	S	S	S	R	R	R	R	R	R	S	R	R	R	R	R	0.75
*E. vulneralis* A1a	R	R	R	R	R	R	R	R	R	R	R	R	R	I	I	I	0.81
*E. vulneralis* B3a	R	S	R	R	S	R	R	R	R	R	S	R	R	R	R	R	0.81
*E. vulneralis* B1a	R	R	R	R	R	R	R	R	R	R	R	R	R	I	I	I	0.81
*S.* Paratyphi B1c	R	R	I	R	S	R	S	R	R	S	S	S	R	I	I	I	0.43
*S.* Paratyphi A1a	R	S	S	R	R	R	R	R	R	R	R	R	R	I	I	I	0.68

S=Susceptible, R=Resistant, I=Intermediate, SXT=Cotrimoxazole, AMC=Amoxycillin-clavulanic acid, AMX = Amoxycillin, CAZ=Ceftazidime, CFP=Cefoperazone, CIP=Ciprofloxacin, CTX=Cefotaxime, CXM=Cefuroxime, ERY=Erythromycin, GEN=Gentamycin, IMP=Imipenem. KAN=Kanamycin, NIF=Nitrofurantoin, OX=Oxacillin, PEN=Penicillin, TE = Tetracycline. For MAR index calculation, bacteria with intermediate resistance were considered as susceptible.

S=Susceptible, R=Resistant, I=Intermediate, SXT=Cotrimoxazole, AMC=Amoxycillin-clavulanic acid, AMX = Amoxycillin, CAZ=Ceftazidime, CFP=Cefoperazone, CIP=Ciprofloxacin, CTX=Cefotaxime, CXM=Cefuroxime, ERY=Erythromycin, GEN=Gentamycin, IMP=Imipenem. KAN=Kanamycin, NIF=Nitrofurantoin, OX=Oxacillin, PEN=Penicillin, TE = Tetracycline. For MAR index calculation, bacteria with intermediate resistance were considered as susceptible.

Regarding the resistance profile of the pathogens to the different antibiotics, it appears from Table 3 that the pathogens tested were resistant to at least three antibiotics. An explanation to this observation could be abuse use of antibiotics in fish farming activities and the non-respect of the withdrawal periods of farmed fishes. As observed in Table 3, the MAR indexes against the different pathogens tested in this study range from 0.18 (*E. coli* A1c, *E. coli* B2d and *Staphylococcus* spp. A1a) to 0.81 (*E. vulneralis* A1a, *E. vulneralis* B3a and *E. vulneralis* B1a). They were no correlation between a bacterial species and the resistance to specific antibiotics.

Independently of the pathogens, the lowest resistances were recorded with penicillin (9.09 %), oxacillin (11.36 %), erythromycin (13.63 %) and gentamycin (15.90 %). However, 97.72 % of pathogens were resistant to cefuroxime, 93.18 % to amoxicillin-clavulanic acid, 93.18 % to ceftazidime, 77.27 % to cefoperazone, 75 % to kanamycin, and 75 % to cefotaxime.

### 3.6 Antimicrobial activity of the spice formulations’ extracts

Table 4 presents the different diameters of inhibition obtained from the aqueous extracts of the different spices’ formulations tested on multi antibiotics resistant pathogens previously isolated from raw and braised fish samples. Globally, the different extracts of spices’ formulations were active against the tested pathogens. Amongst the different formulations, the one containing clove as the differential spice (formulation B) was the most active independently of the pathogens. The highest inhibition diameter was recorded with the extract from that formulation against the isolate *S. aureus* C1a (15.50 ± 0.70 mm). Although less active than the formulation B, formulation A with prekeses as differential spice was more active than formulation C with mbongo as the differential spice. The lowest inhibition diameters were generally observed with formulation C.

**Table 4:** Inhibition diameters (mm) against several pathogens of the aqueous extracts from raw and heated (100^o^C/30 min) formulations of spices used as accompanying soup for braised fish sold in the city of Bangangté.

Strains	Raw			Heated (100^o^C/30 min)		
	Formulation A	Formulation B	Formulation C	Formulation A	Formulation B	Formulation C
*E. coli* A1a	2.02 ± 0.10^b^	4.50 ± 0.70^c^	4.00 ± 0.41^c^	1.50 ± 0.70^b^	2.00 ± 0.10^b^	0.50 ± 0.70^a^
*E. coli* A1c	4.40 ± 0.00^c^	6.50 ± 0.20^d^	3.00 ± 0.00^b^	0.50 ± 0.18^a^	0.90 ± 0.30^a^	0.70 ± 0.20^a^
*E. coli* A3a	6.50 ± 0.70^b^	7.00 ± 0.00^c^	0.00 ± 0.00^a^	0.00 ± 0.00^a^	0.00 ± 0.00^a^	0.00 ± 0.00^a^
*E. coli* A3b	5.90 ± 0.80^d^	8.50 ± 0.70^e^	4.70 ± 0.30^c^	3.50 ± 0.70^b^	5.00 ± 0.71^c^	0.00 ± 0.00^b^
*E. coli* A3c	5.50 ± 0.70^d^	9.50 ± 0.70^e^	9.50 ± 0.70^c^	0.00 ± 0.00^a^	1.50 ± 0.70^b^	0.00 ± 0.00^a^
*E. coli* A2c	4.00 ± 0.22^d^	5.50 ± 0.70^e^	5.00 ± 0.15^e^	1.40 ± 0.04^b^	2.01 ± 0.10^c^	0.30 ± 0.01^a^
*E. coli* A2d	6.00 ± 0.12^d^	6.42 ± 0.10^d^	6.50 ± 0.70^d^	2.50 ± 0.70^b^	3.50 ± 0.12^c^	1.97 ± 0.01^a^
*E. coli* B1a	4.90 ± 0.60^d^	6.50 ± 0.30^e^	4.50 ± 0.22^d^	1.10 ± 0.11^b^	3.00 ± 0.33^c^	0.50 ± 0.10^a^
*E. coli* B1b	3.00 ± 0.10^d^	6.04 ± 0.15^e^	3.30 ± 0.44^d^	1.53 ± 0.70^b^	2.30 ± 0.10^b^	1.50 ± 0.10^a^
*E. coli* B2c	6.50 ± 0.70^d^	7.80 ± 0.44^e^	6.50 ± 0.21^d^	3.20 ± 0.10^b^	4.00 ± 0.40^c^	0.50 ± 0.02^a^
*E. coli* B2d	5.00 ± 0.30^e^	7.02 ± 0.08^f^	4.00 ± 0.16^d^	2.22 ± 0.41^b^	3.05 ± 0.02^c^	0.00 ± 0.00^a^
*E. vulneralis* A1a	3.80 ± 0.10^e^	4.00 ± 0.20^e^	2.60 ± 0.16^d^	0.70 ± 0.03^b^	0.98 ± 0.10^c^	0.00 ± 0.00^a^
*E. vulneralis* B1a	4.00 ± 0.50^b^	6.50 ± 0.70^d^	5.06 ± 0.41^c^	0.00 ± 0.00^a^	0.00 ± 0.00^a^	0.00 ± 0.00^a^
*E. vulneralis* B3a	4.70 ± 0.30^d^	5.50 ± 0.10^e^	3.90 ± 0.70^d^	1.08 ± 0.10^b^	2.00 ± 0.50^c^	0.50 ± 0.10^a^
*S.* Typhi A1c	7.40 ± 0.01^e^	9.00 ± 0.44^f^	6.00 ± 0.00^d^	2.90 ± 0.05^b^	4.00 ± 0.01^c^	1.70 ± 0.20^a^
*S.* Typhi A1d	6.05 ± 0.05^d^	7.09 ± 0.14^e^	4.00 ± 0.10^b^	4.10 ± 0.03^b^	5.30 ± 0.00^c^	1.20 ± 0.07^a^
*S.* Typhi A3b	7.50 ± 0.12^d^	8.00 ± 0.00^e^	5.00 ± 0.00^c^	4.04 ± 0.20^b^	5.10 ± 0.50^c^	3.50 ± 0.70^a^
*S.* Typhi A3a	5.00 ± 0.50^d^	7.50 ± 0.70^e^	4.10 ± 0.08^c^	2.00 ± 1.41^b^	4.00 ± 0.20^c^	1.00 ± 0.00^a^
*S.* Typhi A3c	5.00 ± 0.30^d^	6.50 ± 0.10^e^	3.00 ± 0.20^c^	2.00 ± 0.41^b^	3.80 ± 0.10^c^	1.90 ± 0.07^a^
*S.* Typhi A2a	7.00 ± 1.41^e^	8.00 ± 0.41^f^	6.50 ± 0.70^d^	2.80 ± 0.20^b^	3.04 ± 0.40^b^	1.10 ± 0.02^a^

Strains	Raw			Heated (100^o^C/30 min)		
	Formulation A	Formulation B	Formulation C	Formulation A	Formulation B	Formulation C
*S.* Typhi A2b	7.50 ± 0.70^c^	8.15 ± 0.30^c^	5.00 ± 0.00^b^	5.50 ± 0.70^b^	5.10 ± 0.30^b^	3.70 ± 0.11^a^
*S.* Typhi A2c	8.22 ± 0.31^d^	8.50 ± 0.10^d^	6.50 ± 0.12^c^	4.10 ± 0.70^ab^	4.85 ± 0.10^b^	3.50 ± 0.70^a^
*S.* Typhi A2d	7.50 ± 0.70^e^	7.80 ± 0.00^d^	6.00 ± 0.00^d^	2.00 ± 0.10^b^	2.70 ± 0.01^c^	0.50 ± 0.01^a^
*S.* Typhi B1c	6.75 ± 0.00^d^	8.20 ± 0.10^e^	5.00 ± 1.44^d^	2.00 ± 0.44^b^	3.00 ± 0.30^c^	1.70 ± 0.10^a^
*S.* Typhi B1d	6.00 ± 0.50^c^	8.00 ± 0.00^d^	5.00 ± 1.44^c^	2.00 ± 0.41^b^	4.20 ± 0.80^c^	0.90 ± 0.10^a^
*S.* Typhi B2c	8.00 ± 0.00^e^	8.50 ± 0.17^f^	6.50 ± 0.12^d^	4.05 ± 0.21^b^	5.97 ± 0.18^c^	2.50 ± 0.72^a^
*S.* Typhi B2d	6.50 ± 0.70^d^	7.53 ± 0.01^e^	4.00 ± 0.20^c^	2.83 ± 0.30^b^	3.76 ± 0.40^c^	1.50 ± 0.07^a^
*S.* Typhi C1a	7.50 ± 0.30^d^	9.60 ± 0.11^e^	4.70 ± 0.70^b^	4.30 ± 0.10^b^	6.70 ± 0.00^c^	2.72 ± 0.41^a^
*S.* Typhi C1c	7.20 ± 0.80^d^	8.70 ± 0.30^e^	4.90 ± 0.27^b^	4.55 ± 0.17^b^	6.25 ± 0.10c	2.80 ± 0.18^a^
*S.* Typhi C1b	7.70 ± 0.10^d^	9.40 ± 0.12^c^	4.40 ± 0.14^b^	4.62 ± 0.53^b^	6.70 ± 0.00c	2.33 ± 0.20^a^
*S.* Paratyphi A1a	6.30 ± 0.10^a^	8.50 ± 0.20^b^	6.50 ± 0.70^b^	4.00 ± 0.14^b^	5.50 ± 0.23^b^	0.00 ± 0.00^a^
*S.* Paratyphi B1c	4.00 ± 0.50^a^	7.10 ± 0.40^b^	6.60 ± 0.12^b^	2.00 ± 0.54^b^	3.70 ± 0.10^b^	0.00 ± 0.00^a^
*S. aureus* B1a	13.00 ± 0.10^d^	14.00 ± 0.70^e^	11.50 ± 0.44^b^	11.50 ± 0.10^b^	12.50 ± 0.15^c^	9.00 ± 1.00^a^
*S. aureus* C1a	13.20 ± 0.20^d^	15.50 ± 0.70^e^	10.00 ± 0.10^b^	9.50 ± 0.20^a^	12.00 ± 0.70^c^	8.50 ± 1.00^a^
*Staphylococcus* spp. B1c	12.00 ± 0.41^d^	14.50 ± 0.70^e^	10.50 ± 0.12^c^	8.00 ± 0.30^a^	9.80 ± 0.10^b^	7.00 ± 0.60^a^
*Staphylococcus* spp. B1d	10.50 ± 0.70^d^	13.50 ± 0.70^e^	8.00 ± 1.41^bc^	7.50 ± 0.70^b^	9.00 ± 0.50^c^	5.50 ± 0.70^a^
*Staphylococcus* spp. B2b	10.50 ± 0.12^b^	14.20 ± 0.00^d^	11.50 ± 0.12^c^	7.00 ± 0.70^a^	8.00 ± 0.40^a^	7.50 ± 0.50^a^
*Staphylococcus* spp. B2d	13.00 ± 0.41^d^	14.50 ± 0.70^e^	12.00 ± 0.41^c^	8.50 ± 0.10^b^	8.90 ± 0.50^b^	7.50 ± 0.20^a^
*Staphylococcus* spp. A1a	12.00 ± 0.00^d^	13.50 ± 0.70^e^	9.00 ± 0.00^b^	9.00 ± 0.50^b^	11.00 ± 0.44^c^	7.00 ± 0.10^a^
*Staphylococcus* spp. A1b	13.00 ± 0.10^d^	14.50 ± 0.70^e^	9.00 ± 1.44^b^	10.40 ± 0.00^b^	11.50 ± 0.70^c^	6.50 ± 1.00^a^
*Staphylococcus* spp. A1d	10.50 ± 0.70^b^	12.00 ± 0.00^c^	6.50 ± 0.70^a^	6.00 ± 0.00^a^	10.00 ± 1.00^b^	6.00 ± 0.40^a^
*Staphylococcus* spp. A3a	12.00 ±0.00^d^	14.00 ± 1.44^e^	10.00 ± 0.44^c^	8.00 ± 0.10^b^	8.50 ± 0.50^b^	5.00 ± 0.30^a^
*Staphylococcus* spp. A3b	11.00 ± 0.00^c^	12.50 ± 0.70^d^	8.50 ± 0.70^b^	7.50 ± 0.70^b^	7.00 ± 1.00^b^	4.00 ± 0.20^a^
*Staphylococcus* spp. A3c	10.00 ± 0.50^c^	10.80 ± 0.44^c^	10.00 ± 0.35^c^	8.00 ± 0.30^b^	8.00 ± 0.50^b^	5.00 ± 0.30^a^

Tests were performed in triplicate. Values are means ± standard deviation. Values with different superscript letters within the same raw are significantly different at p < 0.05.

Tests were performed in triplicate. Values are means ± standard deviation. Values with different superscript letters within the same raw are significantly different at p < 0.05.

The susceptibility of the tested pathogens to the different extracts was significantly different (p < 0.05). The most resistant pathogens to extract from formulation A was the strain *E. coli* A1a (2.02 mm) while the most sensitive strains were *S. aureus* B1a and *Staphylococcus* spp. B2d with inhibition diameters of 13 mm each. With extract from formulation B, *S. aureus* C1a was the most sensitive strain (15.50 ± 0.70 mm) and *E. vulneralis* A1a the most resistant (4.00 ± 0.20 mm). For the extract from formulation C, the most resistant strain was *E. coli* A3a (0 mm) and the most sensitive one was *Staphylococcus* spp. B2d (12.00 ± 0.41 mm).

With regards to the heat treatment (cooking process) applied to the formulations, it clearly appears that, the practice leads to a significant (p < 0.05) reduction of the antimicrobial activity of the extracts independently of the tested pathogens. Amongst the extracts from the different heated formulations, the one from formulation B with clove as differential spice has also scored the highest inhibition diameters independently of the tested pathogens. While the extracts from the heated formulation C showed the lowest inhibition diameters that were null and void against some pathogens such as *E. coli* A3a, *E. coli* A3b, *E. coli* A3c, *E. coli* B2d, *E. vulneralis* A1a, *E. vulneralis* B1a, *S.* Paratyphi A1a and *S.* Paratyphi B1c.

Considering the pathogens, it appears from Table 4 that Gram-negative bacteria were generally more resistant to the different extracts independently of the formulations and the heating process.

## 4 Discussion

In this study, we found that the braising fish activity was mainly carried out by women. A similar observation was made by Maffouo et al. [29] with braised fish sold in the city of Yaoundé. This can be explained by the fact that in sub-Saharan Africa and particularly in Cameroon, the informal sector’s involving cooking practices are mainly dominated by women as it requires a relatively small capital base, allows women to do household chores such as child care besides vending [44]. This observation justifies their involvement in activities requiring low capital base in order to supplement their husbands’ lower wages. The main source of fish observed in the city of Bangangté were fish farms. This results differs to those reported by Maffouo et al. [29] where fish were mainly originated from shops and markets. The difference might arise from the climatic conditions of the city of Bangangté that offer a suitable environment for fish farming [7].

The main focus of the survey carried out in this study was spices formulation. It was found that 13 different spices were used by fish vendors as the constituents for the formulation of the spicy soup. Amongst the different spices, the most used by all fish vendors were onions (100 %) followed by white pepper (96 %). This results is consistent with findings of Maffouo et al. [29]. We noticed that, they are three different formulations of spicy soup with clove, mbongo and prekeses as the distinctive ingredients. The formulated spicy soups display two different color corresponding to the way that they were cooked or not. This difference could be attributed to the heat treatment as during that process, some non-enzymatic browning reactions such as the reaction of Maillard occurs between the constituents of spices, leading to the formulation of brown compounds [45,46].

After identifying the spicy soup preparation processes, raw and braised fish were sampled, pathogens were isolated and identified at phenotypic level. The results obtained shows that they belonged to six species *Escherichia coli, Escherichia vulneralis, Salmonella* Typhi*: Salmonella* Paratyphi A*, Staphylococcus aureus* and *Staphylococcus* spp. This microbial diversity might arise from the diverse potential source of contamination of fish during the farming such as animal, household and agricultural wastes as well as the sewage effluents. This results in accordance with findings of Tsafack et al. [6]. The dominant species were *E. coli* and *S.* Typhi while few proportions of *E. vulneralis*, *S.* Paratyphi, *S. aureus* and *Staphylococcus* spp., were identified. This result is different to those reported by Tsafack et al. [6] in Cameroon and Sichewo et al. [47] in Zimbabwe. These authors reported *S. aureus* as the most abundant microbial species in fish.

The antibiotic resistance profile of the different isolates was assessed. Globally, the most active antibiotics against all the tested pathogens were gentamycin, imipenem, ciprofloxacin and amoxiclave. The action mechanisms of these antibiotics against pathogens could explain their efficacy. Indeed, the antibacterial mechanism of ciprofloxacin and imipenem involves the inhibition of the DNA gyrase and topoisomerase IV, enzymes responsible of the replication and transcription of bacterial DNA [48]. Moreover, imipenem also acted by modifying the membrane permeability or interfering with the cell wall formation, leading to loss of integrity, leakage of cytoplasmic constituents, and cell death [49]. The highest activity recorded with gentamycin against all the tested pathogens might be ascribed to its antibacterial action mechanism. Indeed, gentamycin act by blocking the synthesis of protein through a binding to the 30S RNA subunit, leading to the dead of bacteria [50]. Imipenem and gentamycin were also reported as displaying high sensitivity against both Gram positive and negative bacteria by Barber et al. [51]. Pahane et al. [7] evidenced the high sensitivity to gentamycin and imipenem of bacteria isolated from braised products (fish and chicken) sold in the city of Bangangté, Cameroon.

The pathogens tested showed resistance to at least three antibiotics. An explanation to this observation could be abuse use of antibiotics in fish farming activities and the non-respect of the withdrawal periods of farmed fishes. Subjective administration and dosage of antibiotics by fish farmers in Cameroon was reported by Wadoum et al. [52]. An investigation carried by Ntsama et al. [1] on fish farming activities in several regions of Cameroon reported an abuse use of antibiotics without referring to veterinarian prescription by 75 % of fish farmers. Besides, antibiotics are administrated to fish through dispersing in fish ponds. These fish ponds therefore become the reservoir of antibiotic resistant bacteria. A similar observation was made by Fakorede et al. [53]. Tsafack et al. [6] also reported the presence of multiple antibiotic resistant bacteria in fish flesh, fish meal, water and mood from ponds of a fish farming site in the city of Yaoundé, Cameroon. Elsewhere, the development of antibiotic resistance was associated to the irresponsible use of antibiotics and their widespread excessive dispensing [22].

MAR indexes were also calculated to demonstrate the resistance of the pathogens to antibiotics. All the pathogens were multi-resistant with MAR indexes ranging from 0.18 (*E. coli* A1c, *E. coli* B2d and *Staphylococcus* spp. A1a) to 0.81 (*E. vulneralis* A1a, *E. vulneralis* B3a and *E. vulneralis* B1a). The possible resistance mechanisms to these antibiotics could be: (i) the enzymatic degradation of antibiotics, (ii) the horizontal transfer of resistant genes, (iii) a mutation leading to the modification of antibiotic targets, (iv) the hindering of antibiotics’ entry into the cell or elimination of antibiotics present in the cell using the efflux pump [21]. β-lactamases of class A were also reported as the enzyme produced by some bacteria and which are responsible for their high resistance to some antibiotics like ampicillin and amoxicillin [22]. Some enterobacteria harbored AmpC mutations which confer to these latters, the ability to resist to ceftazidime [54]. Modification of bacterial cell permeability through expression of efflux pumps or modification in the porin genes was reported as a resistance mechanism to ceftazidime [54]. Pahane et al. [7] and Tsafack et al. [6] reported the presence of pathogens with high MAR indexes (greater than 0.2) in fish flesh and braised fish. The MAR indexes recorded in this study suggests that more attention should be pay by the government on the monitoring of the use of antibiotics by farmers as well as their sensitization on the harmful effects of abuse use of antibiotics with as targets the bioaccumulation of antibiotic residues in fish flesh and the possible transfer of the resistance genes to human pathogens leading to a continuous increase of the resistance phenomena.

According to Krumperman [42], MAR index value greater than 0.2 indicates that the bacteria tested were isolated from high-risk sources. In this study, except *E. coli* A1c, *E. coli* B2d and *Staphylococcus* spp. A1a, almost bacteria tested show MAR index greater than 0.2. This result suggests that fish farmed in the city of Bangangté could be considered as at high risk of multiple antibiotic resistances. Similar observations were made by Pahane et al. [7] with braised products sold in the city of Bangangté, Cameroon and by Tsafack et al. [6] with fish farmed in the city of Yaoundé, Cameroon.

Globally, this study evidenced that the pathogens were MDR. More insights regarding the antibiotic resistance mechanisms of the pathogens should be obtained through characterization of the isolates with advanced genomic techniques like whole genome sequencing.

In view of proposing alternative to antibiotics for which the pathogens have shown high resistance, aqueous extracts of the different spice formulations were prepared and tested. Interesting results were obtained as the extracts were active against almost tested pathogens. The observed activity could arise from the presence in these spices of bioactive compounds endowed with antimicrobial properties such allicin, phenolic compounds, flavonoids, tannins, terpenoids, phytates and alkaloids [55]. Indeed, allicin has a broad antimicrobial activities against both Gram-positive and -negative bacteria, including those which present antibiotic resistance. It reacts with thiol groups of bacterial proteins and forms *S*-allylmercapto adducts. Hence, thiol groups are oxidated leading to inhibition of many catabolically important enzymes [56]. Phenolic compounds also interact with microbial enzymes involved in the biosynthesis of amino acids [57].

Amongst the different formulations, the one containing clove as the differential spice (formulation B) was the most active independently of the pathogens. This could be ascribed to the presence in clove of compounds endowed with pronounced antimicrobial activity such as eugenol, phenyl propanoid, beta caryophyllene [58]. Although less active than the formulation B, formulation A with prekeses as differential spice was more active than formulation C with mbongo as the differential spice. The weak antimicrobial activity of the formulation C could result from the phytochemical composition of mbongo. Indeed, mbongo contained less compounds with high antimicrobial activity compared to prekeses and clove [59,60].

Globally, it was noticed a great variability in the antimicrobial activity according to the spice formulations and the tested pathogens. Indeed, the pathogens displayed different resistance patterns to the spices’ formulations. These observations could be explained by the content and the composition of bioactive compounds that varies from one formulation to another; and also, to the response of bacteria to antimicrobial compounds that varies from one strain to another [61]. A variation of the antimicrobial mechanism of plant extracts from one bacterium to another was highlighted in the literature [62].

Compared with data reported in the literature for individual spice included in the different formulations, it appears that the antimicrobial activity decreases in the mixture. This observation might arise from the fact that when spices are mixed at different proportions, their activity can be synergistic or not, with as consequence a reduction of the antimicrobial potential. Besides, the insolubility of some bioactive compounds (phenolic compounds, flavonoids, ect.) in water used as extraction solvent could also justify the low antimicrobial activity of the formulations. Al Farraj et al. [55] also highlighted that water cannot extract almost bioactive compounds present in plants.

The heat treatment applied to the formulation of spices during its preparation process has significantly decreased the antimicrobial activity of the extracts independent of the tested pathogens. This could be explained by the fact that, during the heat treatment (100 °C/30 min) some bioactive compounds of the extracts are denatured or interacted, thus leading to a loose of their antimicrobial activity. Indeed, Ginovyan [63] reported that, during heating, some nutrients (proteins, carbohydrates, lipids, vitamins etc.) and bioactive compounds present in the extracts might interact leading to the formation of new ones for which the antimicrobial activity could be increased, reduced or nil.

Considering the pathogens, Gram-negative bacteria were more resistant to the different extracts independent of the formulations or the heating process. This could arise from the outer membrane of Gram-negative bacteria that acted as effective barrier against antimicrobial compounds [64]. A similar observation was also noticed by Mouafo et al. [41] with extracts from two plants (*Millettia laurentii* De Wild and *Lophira alata* Banks ex C. F. Gaertn).

Globally, there was no correlation between the MAR index of the pathogens and their sensitivity to the extracts from the different formulations of spices. This observation highlights that the genes and the metabolic pathway involved in the resistance mechanism against antibiotics are different to those against spices’ extracts. Hence, it therefore appears interesting to identify the antimicrobial mechanism of the spices’ extracts and assess their synergetic or antagonistic effect on the modulation of resistance to the different antibiotics.

## 5 Conclusion

This study demonstrates that thirty spices are used to prepare the spicy accompanying soup of braised fish sold in the city of Bangangté. The spicy accompanying soup is served to consumers either raw or cooked at 100 °C for 30 min. Pathogens belonging to six bacterial species *Escherichia coli, Escherichia vulneralis, Salmonella* Typhi*: Salmonella* Paratyphi A*, Staphylococcus aureus* and *Staphylococcus* spp*.* were identified among from raw and braised fish sold in the city of Bangangté, Camerooon. These pathogens were multi resistant to several commonly used antibiotics thus suggesting their poor and abusive usage in the farming practices. The aqueous extracts from the three spicy soup formulations prepared using two processes (uncooked, and cooked at 100 °C/30 min) display antimicrobial activity against the MDR bacteria. Their use in combination with antibiotics might modulate their effects and thus reduce the multidrug resistance of bacteria. For that, further characterization of compounds involved in the antimicrobial activity as well as their antimicrobial mechanisms should be investigated.

## CRediT authorship contribution statement

**Hippolyte Tene Mouafo:** Writing – review & editing, Validation, Supervision, Software, Project administration, Investigation, Conceptualization. **Majeste Mbiada Pahane:** Writing – review & editing, Visualization, Validation, Supervision, Resources, Methodology, Formal analysis, Data curation, Conceptualization. **Paul Alain Nana:** Visualization, Validation, Software, Data curation. **Hermes Tsabet:** Software, Methodology, Investigation, Data curation. **Alphonse Tegang Sokamte:** Validation, Resources, Conceptualization. **Thierry Ngangmou Noumo:** Software, Methodology, Conceptualization. **Ingrid Cecile Djuikoue:** Supervision, Project administration, Conceptualization. **Agbor Michael Ashu:** Supervision. **François Tchoumbougnang:** Supervision.

## Data availability statement

Data will be made available on request.

## Funding statement

This research did not receive any specific grant from funding agencies in the commercial, public, or not-for-profit sectors.

## Declaration of competing interest

The authors declare that they have no known competing financial interests or personal relationships that could have appeared to influence the work reported in this paper.
